# Supplementary material for: Breast Cancer Risk Assessment Tools for Stratifying Women into Risk Groups: A Systematic Review
Source: Cancers (Basel). 2023 Feb 9;15(4):1124. doi: 10.3390/cancers15041124 (PMC9953796; doi:10.3390/cancers15041124)
Supplement: Supplementary file 1 [file cancers-15-01124-s001.zip › List of excluded studies L1.pdf]

List S1: 78 articles excluded from the review at full text screening stage by reason(s) for exclusion (S indicates snowballed studies; PROSPERO CRD record numbers provided)

Ineligible outcome metric (n=24)

E/O not reported by risk category (only overall): Anothaisintawee 2012 (PMID: 22076477); Banegas 2011 (http://dx.doi.org/10.1158/1538-7445.AM2011-3717); Clift 2020 (CRD42020216681); Hung 2019 (PMID: 31308460); Kurian 2021 (PMID: 34228814); Li 2020 (CRD42020202570); MacInnis 2019 (PMID: 31584660); 2012 (PMID: 22037780); Pfeiffer 2013 (PMID: 23935463); Schonfeld 2010 (PMID: 20368565); Stegeman 2012 (PMID: 22841151); Tice 2019 (S) [PMID: 30796654]; Viallon 2009 (S) [PMID: 19156698]; Wang 2018 (PMID: 29534738)

No outcome metric of interest: Brentnall 2015 (PMID: 25318454); Cornelius-Schecter 2021 (CRD42021256206); Fung 2019 (PMID: 30563826); Mbuya-Bienge 2020 (CRD42020198930); MacInnis 2020 (PMID: 33301022); McCarthy 2013 (PMID: 23474973); Sontag 2011 (PMID: 21178791); Wang 2014 (PMID: 25085581); Wei 2020 (PMID: 33443076); Yala 2021 (PMID: 33504648)

Ineligible study design (n=13):

Case-control: Allman 2021 (PMID: 33481864); Du 2021 (PMID: 33769540); Ji 2021 (PMID: 33739539); Kumar 2020 (PMID: 33163382); Lophatananon 2017 (PMID: 28559460); Ulusoy 2010 (PMID: 19760030); Vachon 2015 (PMID: 25745020); Wacholder 2010 (S) [PMID: 20237344]

Nested case-control: Eriksson 2017 (PMID: 28288659); Rosner 2021 (PMID: 33277321); Shieh 2016 (PMID: 27565998)

Cross-sectional: Stevanato 2019 (PMID: 31759366)

Case-cohort: Van Veen 2018 (PMID: 29346471)

Ineligible comparator (n=12):

No comparator: Han 2021 (PMID: 34051827); Hughes 2019 (PMID: 32923876); MacInnis 2013 (PMID:23942072); Matsuno 2011 (PMID: 21562243); Nickson 2018 (PMID: 30572910); Pastor-Barriuso 2013 (PMID: 23378108); Petracci 2011 (PMID: 21705679); Rosner 2013 (PMID: 24158759); Schonberg 2016 (PMID: 26625899); Wanders 2018 (S) [PMID: 29720220]; Yala 2019 (PMID: 31063083)

No comparator of interest: Glynn 2019 (PMID: 31015199)

Tool development (n = 11):

Darabi 2012 (S) (PMID: 22314178); Gail 2008 (S) (PMID: 18612136); Gail 2009 (S) [PMID: 19535781]; Gao 2012 (PMID: 23164155), Kerlikowske 2015 (S) [PMID: 25824444]; Kerlikowske 2017 (S) [PMID: 28830497]; Lakeman 2020 (PMID: 32624571); Taghipour 2012 (PMID: 22689090); Tice 2008 (PMID: 18316752); Tice 2015 (PMID: 26282663); Tworoger 2014 (PMID: 25135988)

Ineligible population (n=9):

Internal validation: Dartois 2015 (PMID: 25744293); Glynn 2017 (PMID: 28589369)

High risk population: Evans 2016 (PMID: 27559559); Phillips 2019 (PMID: 31853515); Quante 2012 (PMID: 23127309); Quante 2015 (PMID: 25956172)

Breast cancer patients: Kurian 2009 (PMID: 19336551); Zhu 2020 (PMID: 32461838)

LCIS patients at baseline: Lo 2018 (PMID: 29925933)

Ineligible publication type with not enough detail provided (n=4):

Protocol: Gabrielson 2017 (S) [PMID: 28180256]

Comment: Pepe 2008 (S) [PMID: 18612128]

Review: Pu 2014 (PMID: 24114238)

Letter: Spitz 2009 (S) [PMID: 19903803]

Multiple reasons (n=4):

Inclusion of ineligible study design and/or no risk stratified E/O estimates: Louro 2019 (PMID: 31114019); Roman 2019 (S) [PMID: 31841563]; Vilmun 2020 (PMID: 32361308)

Internal validation or ineligible study design (case-control): Hughes 2021 (PMID: 34036224)

Unable to assess fit of tools (n=1):

Vacek 2011 (PMID: 21604157)

1.3 List of included studies containing additional tools that did not meet criteria for inclusion in data synthesis with reason

Choudhury et al. 2020:
BCRAT v3 applied to the Generations Study (GS) cohort and was the only tool for this cohort not calibrated to this population.

Tyrer-Cuzick v8 applied to the Prostate, Lung, Colorectal and Ovarian Cancer Screening Trial (PLCO) cohort and was the only tool not calibrated to this population.

Jee et al. 2020:
USEA tool, an iCARE-Lit based tool using US-based incidence, mortality and risk factor distributions and European-ancestry RRs and was the only tool completely not calibrated to the Korean Cancer Prevention Study Biobank (KCPS-II) cohort used.

Li et al. 2018:
BCRAT v4 which was the only tool applied to the Women's Health Initiative study (WHI) that was not calibrated to the population

Powell 2014:
Tyrer-Cuzick v6.0.0 which was the only tool applied to the Marin Women's Study (MWS) that was not calibrated to the population

1.5 List of included studies with tools that did not meet selection criteria with reason
Hurson et al. 2021

iCARE-BPC3 and iCARE-BPC3 + PRS for the WGHS cohort: Authors confirmed that part of WGHS validation cohort were used in development of iCARE-BPC3 tool.

Tool containing only polygenic risk score (PRS): ineligible tool

Jantzen 2021
BCRAT + PRS: form of internal validation used on the genetic and clinical based subcohort (single data set split to develop and separately validate tool)

Tyrer-Cuzick + PRS: form of internal validation used on the genetic and clinical based subcohort (single data set split to develop and separately validate tool)

PRS only: ineligible tool

McCarthy 2021:
BCSC tool: E/O not reported by risk category (only overall)

Arrospide et al. 2013:
Barlow tool: 5-year absolute risk projected beyond the period for which the tool was developed (1 year).

1.4 Information obtained via author correspondence:
<u>Tool version:</u>

Husing et al, 2021 for BCRAT

Powell et al, 2014 for BCRAT and BRCAPRO versions (via CancerGene)– provided all the information we requested but still couldn't determine version (See discussion for further details. Note that VF also contacted Cancergene support and university of Texas medical centre genetic research contacts about this who did not reply).

<u>Calibration data:</u>

Choudhury et al, 2020 for supplementary Fig 5a PLCO all tools E/O, 95%CI, observed cases per decile data, no data transformation required.

Husing et al, 2021: Fig 1 for all tools provided predicted, observed LL and UL risk in a form that needed further calculation to get E/O and we used our own formula to calculate 95%CIs per decile and overall. We didn't ask for N per decile so equal distribution per decile assumed).

Hurson et al, 2020: for all tools all cohorts provided predicted, observed, LL and UL risk, E/O, 95%CI per decile in a form that needed some calculation to get observed cases. We calculated overall 95%CIs using our own formulas as well. We didn't ask for N per decile so equal distribution per decile assumed.

Terry et al, 2019: sent 5 and 10y E and O with E/O and 95%CIs and number of women per quantile for each tool. We calculated midpoint percentile for plotting from this.

McCarthy et al, 2020: Fig1 O, E, E/O and 95%CIs provided per decile and we double checked with our own E/O formula. We didn't ask for N per decile so equal distribution per decile assumed and rounded down.

Selection criteria:

Hurson et al, 2020: WGHS inquiry for inclusion of iCARE-BPC3
