# Supplementary material for: Breast Cancer Risk Assessment Tools for Stratifying Women into Risk Groups: A Systematic Review
Source: Cancers (Basel). 2023 Feb 9;15(4):1124. doi: 10.3390/cancers15041124 (PMC9953796; doi:10.3390/cancers15041124)
Supplement: Supplementary file 1 [file cancers-15-01124-s001.zip › Suppl Fig S1.pdf]

Figure S1: Absolute risk calibration and observed rate of incident breast cancer for: A) tool comparisons with and without polygenic risk scores by 5-year risk; B) other risk assessment tool comparisons by 5-year risk; C) risk assessment tool comparisons by 10Hyear risk

A) Risk assessment tool comparisons with and without polygenic risk scores by 5Hyear risk

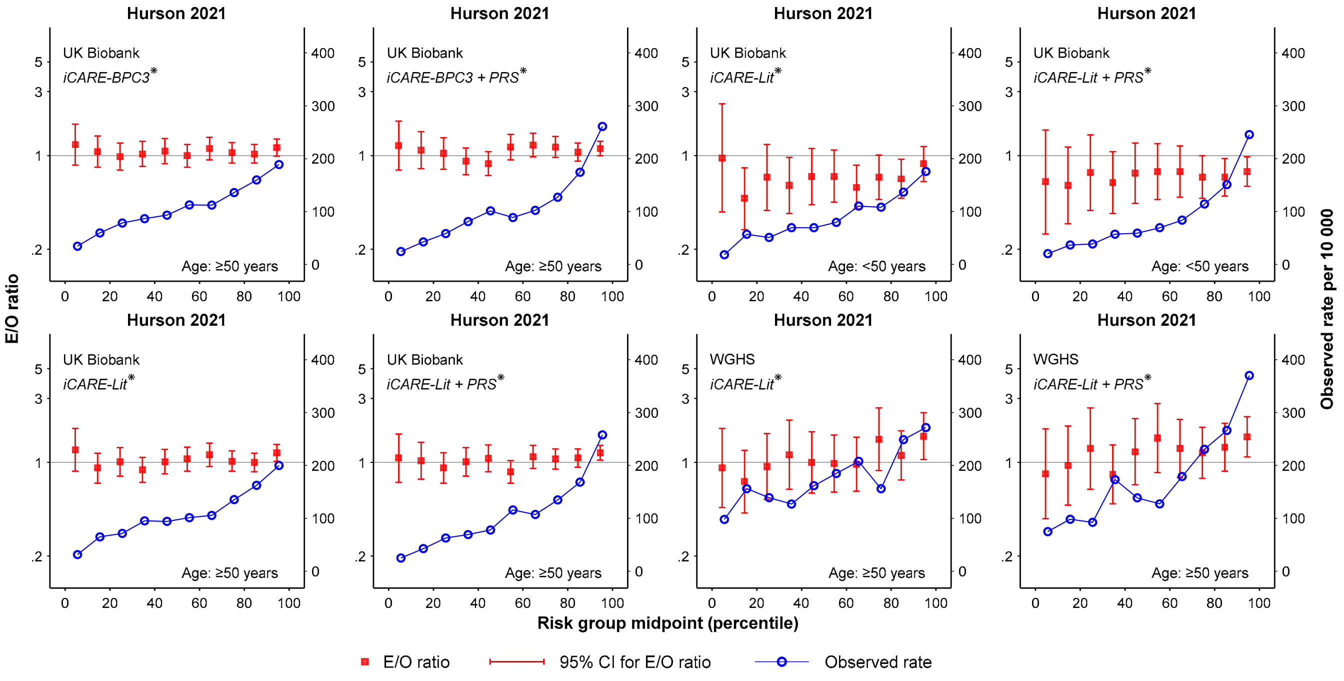

* tools were calibrated to local population. Italicised font indicates risk tool.

Abbreviations: WGHS: Women's Genome Health Study

B) Other risk assessment tool comparisons by 5Hyear risk

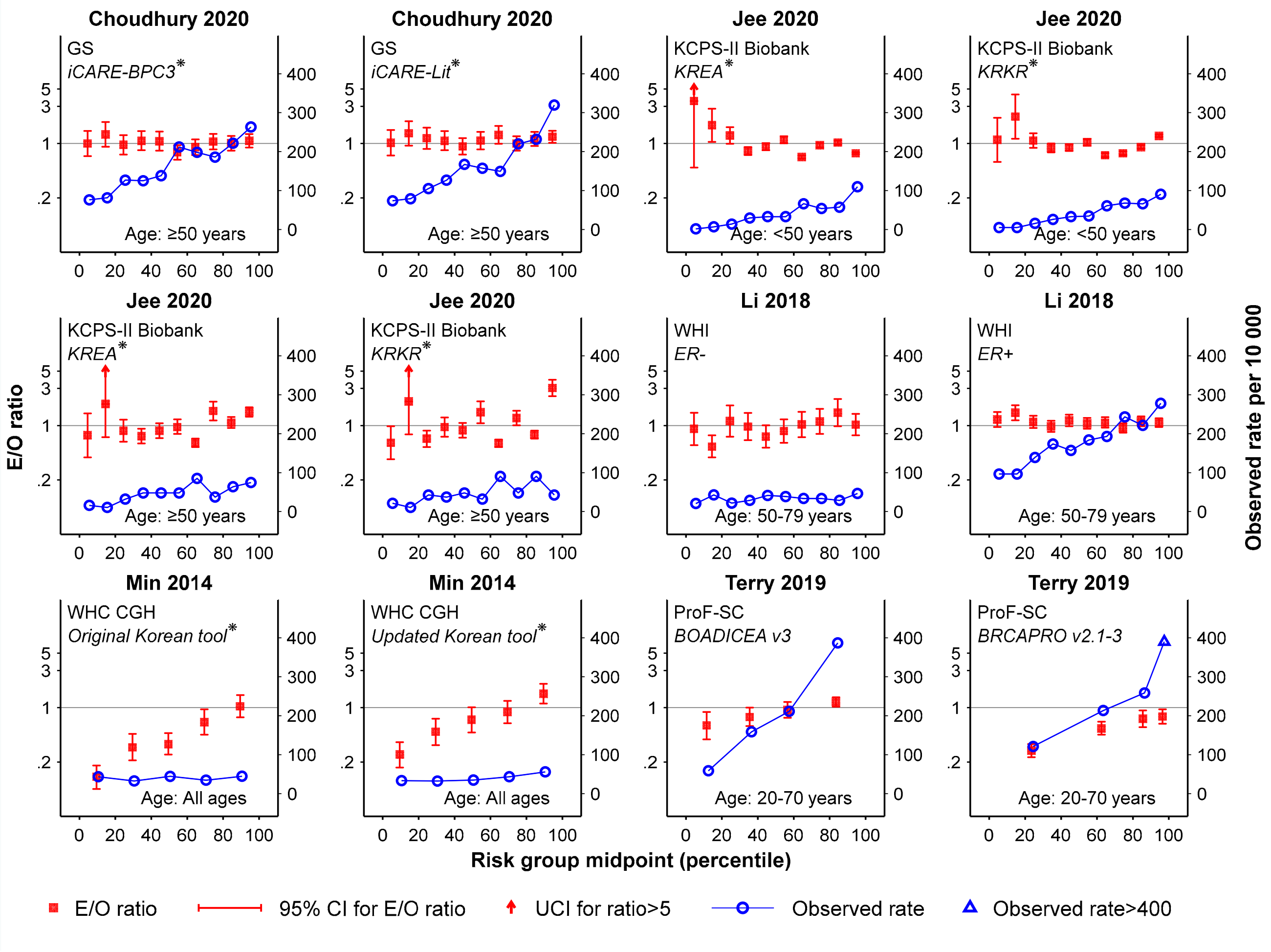

Abbreviations: GS: Generations Study; KCPSHII Biobank: Korean Cancer Prevention StudyHII Biobank; ProFHSC: Breast Cancer Prospective Family Study Cohort; WHC CGH: Women's Healthcare Center of Cheil General Hospital; WHI: Women's Health Initiative

C) Risk assessment tool comparisons by 10Hyear risk

Abbreviations: KPWHBCSC: Kaiser Permanente Washington Breast Cancer Surveillance Consortium registry; ProFHSC: Breast Cancer Prospective Family Study Cohort; SBCSP: Singapore Breast Cancer Screening Project

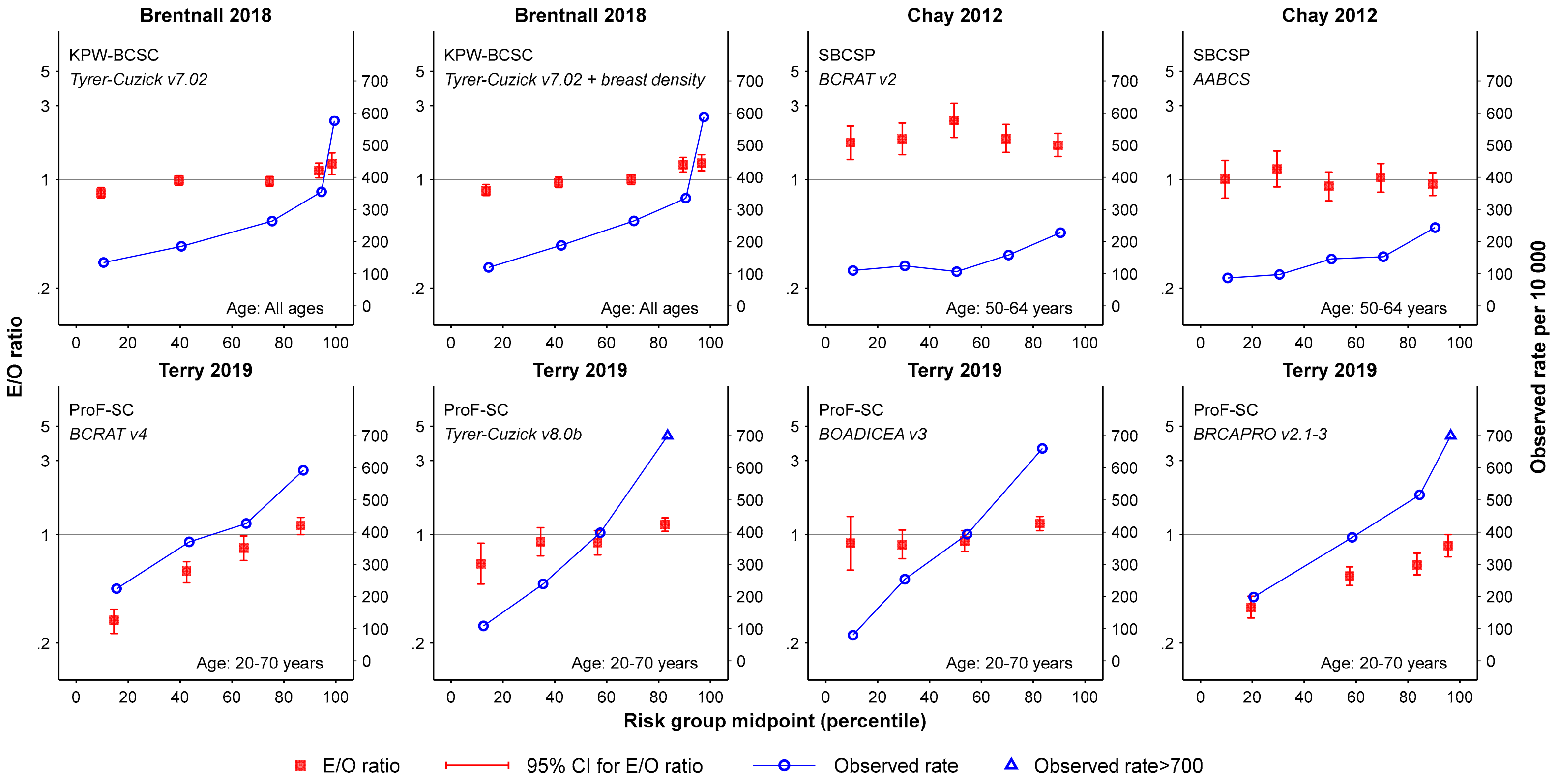

Abbreviations: KPWHBCSC: Kaiser Permanente Washington Breast Cancer Surveillance Consortium registry; ProFHSC: Breast Cancer Prospective Family Study Cohort; SBCSP: Singapore Breast Cancer Screening Project
