# Supplementary material for: Breast Cancer Risk Assessment Tools for Stratifying Women into Risk Groups: A Systematic Review
Source: Cancers (Basel). 2023 Feb 9;15(4):1124. doi: 10.3390/cancers15041124 (PMC9953796; doi:10.3390/cancers15041124)
Supplement: Supplementary file 1 [file cancers-15-01124-s001.zip › Suppl Table S1.pdf]

**Table S1: Search strategy used to identify potentially relevant records in Medline and Embase databases**

Database(s): Embase Classic+Embase 1947 to 2021 June 30, Ovid MEDLINE(R) ALL 1946 to June 30, 2021

| | |
|---|---|
| 1 | exp Breast Neoplasms/ |
| | |
| 2 | (breast adj3 (cancer* or tumour* or tumor* or carcinoma* or neoplasm* or screen*)).tw. |
| 3 | 1 or 2 |
| | |
| 4 | exp risk assessment/ or breast cancer risk assessment tool/ |
| | |
| 5 | ((risk* or susceptib* or predict*) adj6 (tool* or score* or model* or questionnaire* or instrument* or appraisal* or calculation* or calculator* or algorithm*)).tw. |
| | |
| 6 | risk factor* calculat*.tw. |
| | |
| 7 | (risk adj2 (assess* or predict*)).tw. |
| | |
| 8 | (observed* adj4 (expected* or predict* or assigned)).tw. |
| | |
| 9 | observed-to-expected.tw. |
| | |
| 10 | expected-to-observed.tw. |
| | |
| 11 | ("E/O" or "O/E").tw. |
| | |
| 12 | (validat* or calibrat* or area under the curve or c-statistic or AUC or AUROC or receiver operat*).tw. |
| | |
| 13 | (clinical conference or conference or comment or editorial).tw. |
| | |
| 14 | (neoadjuvant or recurrent or recurrence or chemotherapy).tw. |
| | |
| 15 | 4 or 5 or 6 or 7 |
| | |
| 16 | 8 or 9 or 10 or 11 or 12 |
| | |
| 17 | 3 and 15 and 16 |
| | |
| 18 | 17 not 13 |
| | |
| 19 | 18 not 14 |
| | |
| 20 | limit 19 to (english language and humans and yr="2008 -Current") |
| | |
| 21 | remove duplicates from 20 |
