# Supplementary material for: Breast Cancer Risk Assessment Tools for Stratifying Women into Risk Groups: A Systematic Review
Source: Cancers (Basel). 2023 Feb 9;15(4):1124. doi: 10.3390/cancers15041124 (PMC9953796; doi:10.3390/cancers15041124)
Supplement: Supplementary file 1 [file cancers-15-01124-s001.zip › Suppl Table S2_9Feb2023.pdf]

Table S2: Risk predictors within risk assessment tools compared in the studies included in the review ('x': inclusion of predictor data, '-' : absence of predictor data).[a]

Risk assessment tool	Age	Age of menarche	Age of 1st live birth/ FTP	Age of menopause	Parity	Number of FTP	Menopausal status	HRT use	Oral contraceptive use	Breast density	Hysterectomy	BMI or weight	Height	Race and/ or ethnicity	Prior/ no. of breast biopsies	Other pathology	ADH	LCIS	History/ no. of degree relatives assessed	Age of onset of breast cancer	Bilateral breast cancer	Male breast cancer	Ovarian cancer (personal/ familial)	Alcohol intake	Smoking status	Time spent breastfeeding	HRT type/ duration of use	BRCA1/ BRCA2 status	Polygenic SNP score	Study ID
AABCS	x	x	x												x				1st											Chay et al 2012 [32]
AABCS	x	x	x												x				1st											Min et al 2014 [36]
BCRAT v1	x (>35 years)	x	x												x				1st											Arrospide et al 2013 [33]
BCRAT v2	x (>35 years)	x	x												x				1st											Chay et al 2012 [32]
BCRAT v2	x (>35 years)	x	x												x				1st											Min et al 2014 [36]
BCRAT v2-3	x (>35 years)	x	x												-				1st											Powell et al 2014 [30]
BCRAT v3	x (>35 years)	x	x												x		x		1st											Choudhury et al 2020 (PLCO) [35]
BCRAT v3	x (>35 years)	x	x												-		-		1st											Hüsing et al 2020 [37]
BCRAT v3 recalibrated	x (>35 years)	x	x												-		-		1st											Hüsing et al 2020
BCRAT v4	x (>35 years)	x	x											x	x		-		1st											Jantzen et al 2021 [39]
BCRAT v4	x (>35 years)	x	x											x	x		x		1st											McCarthy et al 2020 [38]
BCRAT v4	x (>35 years)	x	x											x	x		-		1st											Terry et al 2019 [31]
BCRmod	x	x	x				x					x							1st					x						Hüsing et al 2020 [29]
BCRmod recalibrated	x	x	x				x					x							1st					x						Hüsing et al 2020

Risk assessment tool	Age	Age of menarche	Age of 1st live birth/ FTP	Age of menopause	Parity	Number of FTP	Menopausal status	HRT use	Oral contraceptive use	Breast density	Hysterectomy	BMI or weight	Height	Race and/ or ethnicity	Prior/ no. of breast biopsies	Other pathology	ADH	LCIS	History/ no. of degree relatives assessed	Age of onset of breast cancer	Bilateral breast cancer	Male breast cancer	Ovarian cancer (personal/ familial)	Alcohol intake	Smoking status	Time spent breastfeeding	HRT type/ duration of use	BRCA1/ BRCA2 status	Polygenic SNP score	Study ID
BOADICEA v3	x													x		x			1st<br>2nd<br>3rd	x	x		x					x		Terry et al 2019 [31]
BRCAPRO v2.1-4	x													x		-		-	Any	x	x		x					-		McCarthy et al 2020 [38]
BRCAPRO v NR	x																	x	1st	x										Powell et al 2014 [30]
BRCAPRO v2.1-3	x													x		x		-	1st<br>2nd<br>3rd	x	x		x					x		Terry et al 2019 [31]
Chen v1	x		x							x		x			x				1st											Arrospide et al 2013 [33]
ER+	x	x	x	x		x	x					x	x											x		x	x			Li et al 2018 [41]
ER-	x	x	x	x		x	x					x	x											x		x	x			Li et al 2018
i-CARE-Lit (≥50)	x	x	x	x	x			x	x			x	x		x				1st					x			x			Choudhury et al 2020 (GS) [35]
i-CARE-Lit (≥50)	x	x	x	x	x			x	x			x	x		x				1st					x			-			Choudhury et al 2020 (PLCO) [35]
i-CARE-Lit (≥50)	x	x	x	x	x			x	x			x	x		-				1st					x			-			Hurson et al 2021 (UK Biobank) [29]
i-CARE-Lit (≥50)	x	x	x	x	x			x	x			x	x		x				1st					x			-			Hurson et al 2021 (WGHS)
i-CARE-Lit + PRS (≥50)	x	x	x	x	x			x	x			x	x		-				1st					x			-		x	Hurson et al 2021 (UK Biobank)
i-CARE-Lit + PRS (≥50)	x	x	x	x	x			x	x			x	x		x				1st					x			-		x	Hurson et al 2021 (WGHS)

Risk assessment tool	Age	Age of menarche	Age of 1st live birth/ FTP	Age of menopause	Parity	Number of FTP	Menopausal status	HRT use	Oral contraceptive use	Breast density	Hysterectomy	BMI or weight	Height	Race and/ or ethnicity	Prior/ no. of breast biopsies	Other pathology	ADH	LCIS	History/ no. of degree relatives assessed	Age of onset of breast cancer	Bilateral breast cancer	Male breast cancer	Ovarian cancer (personal/ familial)	Alcohol intake	Smoking status	Time spent breastfeeding	HRT type/ duration of use	BRCA1/ BRCA2 status	Polygenic SNP score	Study ID
i-CARE-BPC3 (≥50)	x	x	x	x	x		x	x				x							1st					x						Choudhury et al 2020 (GS) [35]
i-CARE-BPC3 (≥50)	x	x	x	x	x		x	x				x							1st					x						Hurson et al 2021 (UK Biobank) [29]
i-CARE-BPC3 + PRS (≥50)	x	x	x	x	x		x	x				x							1st					x					x	Hurson et al 2021 (UK Biobank)
KREA	x	x	x	x	x			x	x			x	x		x				1st					x						Jee et al 2020 [40]
KRKR	x	x	x	x	x			x	x			x	x		x				1st					x						Jee et al 2020
Korean (original)	x		x				x					x			x				1st<br>2nd							x				Min et al 2014 [36]
Korean (updated)	x		x				x					x			x				1st<br>2nd							x				Min et al 2014
TC v7	x	x	x	x	x			x						x	x		x	x	1st<br>2nd<br>3rd				x							McCarthy et al 2020 [38]
TC v7.02	x	x	x	x	x		x					x	x		x	x	x		1st<br>2nd				-							Brentnall et al 2018 [34]
TC v7.02 + BD	x	x	x	x	x		x			x		x	x		x	x	x		1st<br>2nd				-							Brentnall et al 2018
TC v8	x	x	x	x	x		x	x				x	x			-	-	-	1st				-				-			Choudhury et al 2020 (GS)

Risk assessment tool	Age	Age of menarche	Age of 1st live birth/ FTP	Age of menopause	Parity	Number of FTP	Menopausal status	HRT use	Oral contraceptive use	Breast density	Hysterectomy	BMI or weight	Height	Race and/ or ethnicity	Prior/ no. of breast biopsies	Other pathology	ADH	LCIS	History/ no. of degree relatives assessed	Age of onset of breast cancer	Bilateral breast cancer	Male breast cancer	Ovarian cancer (personal/ familial)	Alcohol intake	Smoking status	Time spent breastfeeding	HRT type/ duration of use	BRCA1/ BRCA2 status	Polygenic SNP score	Study ID
TC v8	x	x	x	x	x		x	x				x	x			x	x	x	1st<br>2nd				x				x			Jantzen et al 2021 (CC) [39]
TC v8.0b	x	x	x	x	x	-	-	x	-	x	-	-	-	x	x	-	x	x	1st<br>2nd<br>3rd	-			x				-	-		McCarthy et al 2020 [38]
TC v8.0b	x	x	x	-	x	-	x	x	-	-	-	x	x	-	x	-	-	-	1st<br>2nd	x			x				-	x		Terry et al 2019 [31]

[a] Empty cells indicate predictor not included in tool.

Abbreviations: AABCS = Asian American Breast Cancer Study; ADH = atypical ductal hyperplasia; BCRAT = Breast Cancer Risk Assessment Tool; BCRmod = Breast Cancer Risk tool including modifiable variables; BD = breast density; BMI = body mass index; BOADICEA = Breast and Ovarian Analysis of Disease Incidence and Carrier Estimation Algorithm model; BRCAPRO = BRCA1 probability model; ER+ = Estrogen receptor positive model ; ER- = Estrogen receptor negative model; FTP = full term pregnancy; HRT = hormone replacement therapy; i-CARE- Lit = Individualized Coherent Absolute Risk Estimation-Literature model; i-CARE- BPC3 = Individualized Coherent Absolute Risk Estimation-Breast and Prostate Cancer Consortium model; KREA = recalibrated Korean-European model; KRKR = Korean-based model; LCIS = lobular carcinoma in situ; NR = not reported; PRS = polygenic risk score; TC = Tyrer Cuzick model; v = version.
