# Supplementary material for: Breast Cancer Risk Assessment Tools for Stratifying Women into Risk Groups: A Systematic Review
Source: Cancers (Basel). 2023 Feb 9;15(4):1124. doi: 10.3390/cancers15041124 (PMC9953796; doi:10.3390/cancers15041124)
Supplement: Supplementary file 1 [file cancers-15-01124-s001.zip › Suppl Table S3_9Feb2023.pdf]

Table S3: Detailed assessment of risk of bias of included prognostic risk assessment tool for breast cancer calibration outcomes

Study	RAT	Cohort	Year	Outcome	Participants			Predictors				Outcome							Analysis[a]							Overall
					1.1	1.2	RoB	2.1	2.2	2.3	RoB	3.1	3.2	3.3	3.4	3.5	3.6	RoB	4.1	4.2	4.3	4.4	4.6	4.7	RoB	RoB
Hurson 2021 [29]	iCARE BPC3	UK Biobank	5	Invasive or DCIS	L	L	L	L	L	L	L	L	L	L	L	U	U	U	H	U	L	H	L	L	H	H
Hurson 2021	iCARE BPC3 L PRS	UK Biobank	5	Invasive or DCIS	L	L	L	L	U	L	U	L	L	L	L	U	U	U	H	U	L	H	L	L	H	H
Hurson 2021	iCARE Lit	UK Biobank	5	Invasive or DCIS	L	L	L	L	L	L	L	L	L	L	L	U	U	U	H	U	L	H	L	L	H	H
Hurson 2021	iCARE Lit L PRS	UK Biobank	5	Invasive or DCIS	L	L	L	L	U	L	U	L	L	L	L	U	U	U	H	U	L	H	L	L	H	H
Hurson 2021	iCARE Lit	WGHS	5	Invasive or DCIS	L	L	L	U	U	L	U	L	L	L	U	U	L	U	H	U	L	H	L	L	H	H
Hurson 2021	iCARE Lit L PRS	WGHS	5	Invasive or DCIS	L	L	L	U	U	L	U	L	L	L	U	U	L	U	H	U	L	H	L	L	H	H
Jantzen 2021 [39]	TC v8	CARTaGENE	5	Invasive	L	L	L	L	L	L	L	H	L	L	U	L	L	U	H	L	H	H	L	L	H	H
Jantzen 2021	BCRAT v4	CARTaGENE	5	Invasive	L	L	L	L	L	L	L	H	L	L	U	L	L	U	H	L	H	H	L	L	H	H
McCarthy 2020 [38]	TC v7	NWH	6	Invasive	L	H	H	L	L	L	L	L	U	L	U	U	L	U	H	U	L	L	L	L	H	H
McCarthy 2020	TC v8.0b	NWH	6	Invasive	L	H	H	L	L	L	L	L	U	L	U	U	L	U	H	U	L	L	L	L	H	H
McCarthy 2020	BCRAT v4	NWH	6	Invasive	L	L	L	L	L	L	L	L	U	L	U	U	L	U	H	U	L	U	L	L	H	H
McCarthy 2020	BRCAPRO v2.1H4	NWH	6	Invasive	L	H	H	L	L	L	L	L	U	L	U	U	L	U	H	U	L	L	L	L	H	H
Choudhury 2020 [35]	TC v8	GS	5	Invasive	L	L	L	U	L	U	U	L	U	L	L	U	L	U	H	U	L	U	L	L	H	H
Choudhury 2020	iCARE Lit	GS	5	Invasive	L	L	L	U	L	L	U	L	U	L	L	U	L	U	H	U	L	U	L	L	H	H
Choudhury 2020	iCARE BPC3	GS	5	Invasive	L	L	L	U	L	L	U	L	U	L	L	U	L	U	H	U	L	U	L	L	H	H
Choudhury 2020	BCRAT v3	PLCO	5	Invasive	L	L	L	U	U	L	L	U	U	L	L	U	L	U	H	U	H	H	L	L	H	H

Choudhury 2020	iCARE Lit	PLCO	5	Invasive	L	L	L	U	U	L	L	U	U	L	L	U	L	U	H	U	H	H	L	L	H	H
Hüsing 2020 [37]	BCRAT v3	EPICHGermany	5	Invasive	L	H	H	L	U	L	U	H	L	L	L	U	U	H	H	H	L	H	L	L	H	H
Hüsing 2020	BCRmod	EPICHGermany	5	Invasive	L	L	L	L	U	L	U	H	L	L	L	U	U	H	H	L	L	H	L	L	H	H
Hüsing 2020	BCRAT v3 recalibrated	EPICHGermany	5	Invasive	L	H	H	L	U	L	U	H	L	L	L	U	U	H	H	H	L	H	L	L	H	H
Hüsing 2020	BCRmod recalibrated	EPICHGermany	5	Invasive	L	L	L	L	U	L	U	H	L	L	L	U	U	H	H	L	L	H	L	L	H	H
Jee 2020 [40]	KREA	KCPSHII Biobank	5	Invasive	L	L	L	L	L	L	L	L	L	L	U	U	L	U	H	L	H	U	L	L	H	H
Jee 2020	KRKR	KCPSHII Biobank	5	Invasive	L	L	L	L	L	L	L	L	L	L	U	U	L	U	H	L	H	U	L	L	H	H
Terry 2019 [31]	BCRAT v4	ProFHSC	5	Invasive	L	H	H	L	L	H	H	H	H	L	H	U	U	H	H	U	H	H	L	L	H	H
Terry 2019	BRCAPRO v2.1H3	ProFHSC	5	Invasive	L	L	L	H	U	U	H	H	H	L	H	U	U	H	H	U	H	H	L	L	H	H
Terry 2019	TC v8.0b	ProFHSC	5	Invasive	L	L	L	H	U	U	H	H	H	L	H	U	U	H	H	U	H	H	L	L	H	H
Terry 2019	BOADICEA v3	ProFHSC	5	Invasive	L	L	L	H	U	U	H	H	H	L	H	U	U	H	H	U	H	H	L	L	H	H
Terry 2019	BCRAT v4	ProFHSC	10	Invasive	L	H	H	L	L	H	H	H	H	L	H	U	H	H	L	U	H	H	L	L	H	H
Terry 2019	BRCAPRO v2.1H3	ProFHSC	10	Invasive	L	L	L	H	U	U	H	H	H	L	H	U	H	H	L	U	H	H	L	L	H	H
Terry 2019	TC v8.0b	ProFHSC	10	Invasive	L	L	L	H	U	U	H	H	H	L	H	U	H	H	H	U	H	H	L	L	H	H
Terry 2019	BOADICEA v3	ProFHSC	10	Invasive	L	L	L	H	U	U	H	H	H	L	H	U	H	H	H	U	H	H	L	L	H	H
Brentnall 2018 [34]	TC v7.02	KPWHBCSC	10	Invasive	L	L	L	U	L	H	H	L	U	L	U	U	U	U	L	L	L	H	L	L	H	H
Brentnall 2018	TC v7.02 LBD	KPWHBCSC	10	Invasive	L	L	L	U	L	H	H	L	U	L	U	U	U	U	L	L	L	H	L	L	H	H
Li 2018 [41]	ERH	WHI	5	Invasive	L	L	L	U	L	L	U	H	L	L	L	L	U	H	H	U	H	H	U	L	H	H
Li 2018	ERL	WHI	5	Invasive	L	L	L	U	L	L	U	H	L	L	L	L	U	H	H	U	H	H	U	L	H	H
Min 2014 [36]	BCRAT v2	WHC CGH	5	Invasive	L	H	H	L	L	L	L	L	U	L	U	U	U	U	H	L	H	H	L	L	H	H
Min 2014	AABCS	WHC CGH	5	Invasive	L	H	H	L	L	L	L	L	U	L	U	U	U	U	H	L	H	H	L	L	H	H

Min 2014	Original Korean tool	WHC CGH	5	Invasive	L	H	H	L	L	L	L	L	U	L	U	U	U	U	H	L	H	H	L	L	H	H
Min 2014	Updated Korean tool	WHC CGH	5	Invasive	L	H	H	L	L	L	L	L	U	L	U	U	U	U	H	L	H	H	L	L	H	H
Powell 2014 [30]	BCRAT v2 or 3	MWS	5	Invasive	L	H	H	L	H	U	H	L	L	L	U	L	L	U	H	U	L	H	L	L	H	H
Powell 2014	BRCAPRO v(NR)	MWS	5	Invasive	L	L	L	L	H	H	H	L	L	L	U	L	L	U	H	L	L	H	L	L	H	H
Arrospide 2013 [33]	BCRAT v1	SCHBCSP	5	Invasive	L	L	L	L	L	L	L	H	U	L	U	L	U	H	H	U	H	U	H	H	H	H
Arrospide 2013	Chen v1	SCHBCSP	5	Invasive	L	L	L	H	L	L	H	H	U	L	U	U	U	H	H	H	H	U	H	H	H	H
Chay 2012 [32]	BCRAT v2	SBCSP	5	Invasive	L	L	L	L	L	H	H	L	U	L	U	U	U	U	H	H	U	H	H	L	H	H
Chay 2012	AABCS	SBCSP	5	Invasive	L	L	L	L	L	H	H	L	U	L	U	U	U	U	H	H	U	H	H	L	H	H
Chay 2012	BCRAT v2	SBCSP	10	Invasive	L	L	L	L	L	H	H	L	U	L	U	U	U	U	H	H	U	H	H	L	H	H
Chay 2012	AABCS	SBCSP	10	Invasive	L	L	L	L	L	H	H	L	U	L	U	U	U	U	H	H	U	H	H	L	H	H

[a]Notes: i) different age groups for item 4.1 were not assessed separately (if for example an <50y age group was rated as high risk of bias then the whole item rating was high risk); ii) as studies only reported one breast cancer outcome (either invasive or invasive L DCIS) multiple outcome assessments were not required; iii) Items 4.5, 4.8 and 4.9 omitted as they are signalling questions for model development and not validation;

Key to domain and overall rating: High risk of bias: indicated as ‘H‘; low risk of bias: indicated as L; unclear risk of bias: indicated as ‘U’
